# The Mediating Role of Alexithymia on the Relationship between Intolerance of Uncertainty and Body Dissatisfaction

**DOI:** 10.1192/j.eurpsy.2026.12034

**Published:** 2026-07-31

**Authors:** M. B. Ari, F. Eroglu Ada

**Affiliations:** 1 Psychology, Maltepe University; 2 Psychology, Mimar Sinan Fine Arts University, İstanbul, Türkiye

## Abstract

**Introduction:**

With the increasing prevalence of eating disorders, researchers emphasize the importance of studying body dissatisfaction, one of the risk factors that predicts eating disorders, in the design of prevention/intervention studies. Researches have shown that intolerance of uncertainty increases individuals’ anxiety level, thereby creating a basis for body image-related problems. In addition, high levels of alexithymia, by causing difficulties in identifying and expressing emotions, negatively affect individuals’ perceptions of their bodies. In the literature, both intolerance of uncertainty and alexithymia are associated with body dissatisfaction. According to explanations of emotion regulation theory, individuals with a high level of intolerance to uncertainty may find it difficult to use effective emotion regulation strategies. Difficulties in emotion regulation skills may also lead individuals to seek control over their body image and increase the risk of body dissatisfaction.

**Objectives:**

The present study aims to examine the mediating role of alexithymia on the relationship between intolerance of uncertainty and body dissatisfaction. More specifically, it was hypothesized that individuals with low tolerance for uncertainty would exhibit higher levels of alexithymia, which in turn would lead to greater body dissatisfaction.

**Methods:**

The participants comprise 359 university students recruited through convenience sampling. Adolescents were requested to complete the Intolerance of Uncertainty Scale, Body Satisfaction Scale, Multidimensional Body-Self Relations Questionnaire, Toronto Alexithymia Scale, and a demographic information form. The hypotheses were tested with PROCESS Macro Model 4.

**Results:**

The results revealed that body dissatisfaction was significantly correlated with intolerance of uncertainty, alexithymia, and income level. Furthermore, alexithymia was found to fully mediate the relationship between intolerance of uncertainty and body dissatisfaction, even after controlling for the income level.

**Conclusions:**

The findings of the present study indicate that high levels of intolerance of uncertainty play only an indirect role through alexithymia in increasing body dissatisfaction. It is believed that understanding the factors that pose a risk for body dissatisfaction is of critical importance in treatment processes. Therefore, the present findings are expected to contribute to the literature by enhancing the understanding of the mechanisms associated with body dissatisfaction.

**Disclosure of Interest:**

None Declared

